# Hearing Loss: Self‐Reported Onset and Etiology Among Older Adults in the United States

**DOI:** 10.1002/oto2.146

**Published:** 2024-06-06

**Authors:** Tyler J. Gallagher, Ziphron Russel, Janet S. Choi

**Affiliations:** ^1^ Keck School of Medicine of the University of Southern California Los Angeles California USA; ^2^ University of Southern California Los Angeles California USA; ^3^ Caruso Department of Otolaryngology–Head and Neck Surgery Keck School of Medicine of the University of Southern California Los Angeles California USA

**Keywords:** duration, etiology, hearing loss, older adults

## Abstract

This study investigated self‐reported age of onset and etiology of hearing loss among older adults in the United States. Study cohort included older adult (≥70 years) survey respondents from the 2017 to 2020 National Health and Nutrition Examination Survey (n = 797). Overall, 51.1% [95% confidence interval [CI]: 46.1‐56.1] of older adults self‐reported hearing loss. Among older adults who reported hearing loss, the most reported age of onset was age 70 or older (41.7% [95% CI: 38.1%‐45.3%]), followed by sequentially younger age brackets including ages 60 to 69 years (27.3% [95% CI: 23.6%‐31.3%]) and ages 40 to 59 years (15.7% [95% CI: 12.9%‐19.0%]). The most common etiology of hearing loss was aging (66.3% [95% CI: 60.8%‐71.4%]) followed by loud long‐term noise (30.3% [95% CI: 26.2%‐34.9%]) and loud brief noise (13.8% [95% CI: 10.3%‐18.4%]). Our study describes the most common age of onset and etiologies of hearing loss among a representative sample of United States older adults.

Hearing loss is highly prevalent and has associations with significant health consequences among older adults, including social isolation, depression, cognitive decline, and mortality.^1^, ^2^, ^3^ Furthermore, prior studies have shown that age of onset and etiology of hearing loss can have a differential association with hearing loss outcomes.^4^, ^5^ Understanding the national prevalence of self‐reported age of onset when older adults started noticing hearing changes and etiology of hearing loss has implications in development of preventative and management guidelines.^6^, ^7^, ^8^ This study examines prevalence of self‐reported age of onset and etiology of hearing loss among a nationally representative cohort of older adults in the United States.

## Methods

**Figure 1:**
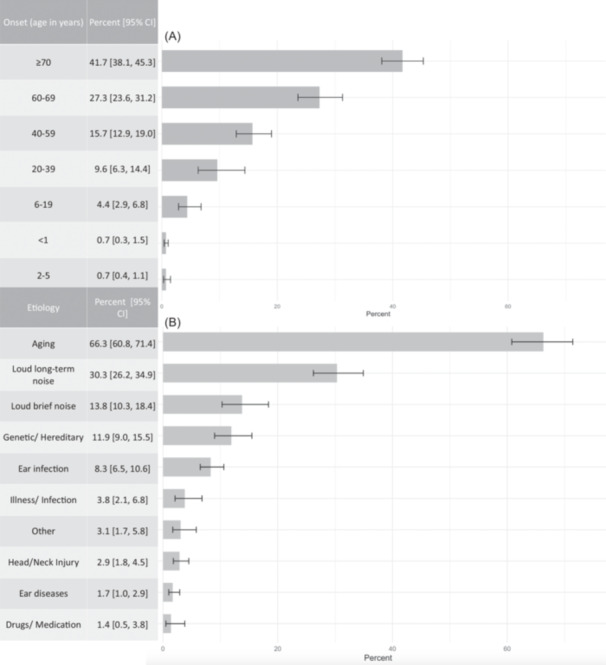
Prevalence of reported (A) Onset and (B) Etiology of hearing loss among older adults, aged 70 years or older, who self‐reported hearing loss in the United States (percent [95% confidence interval]). **Participants were able to select multiple etiologies. Percentages account for percent reporting a particular etiology/onset divided by all individuals of ≥70 years who self‐reported hearing loss.

Study cohort included older adult (≥70 years old) survey respondents from the 2017 to 2020 National Health and Nutrition Examination Survey who self‐reported hearing loss as well as cause and onset of hearing loss (n = 797).^9^ Among a subgroup of participants who completed audiometry (n = 567; 71.1%), all participants had ≥25 decibels of hearing loss in at least 1 frequency of either ear. Specifically, participants were asked “How old were you when you began to have hearing loss?” (onset) and “What are the main causes of your hearing loss?” (etiology). Options for selection are those listed in Figure 1; individuals could select 1 choice for onset and 1 or more for etiology. This study was approved as an exempted study by the University of Southern California Institutional Review Board (UP‐20‐01447). Sample weights were utilized to account for the complex sampling design based on the NHANES analytic guidelines.^10^ Analyses were performed using STATA (v18; STATACorp.).

## Results

Overall, 51.1% [95% CI: 46.1‐56.1] of older United States adults (≥70 years) self‐reported hearing loss. Table 1 describes the weighted demographic and clinical characteristics of this cohort. Among older adults who reported hearing loss, the most commonly reported age of onset was at age 70 or older (41.7% [95% CI: 38.1%‐45.3%]), followed by sequentially younger age brackets including ages 60 to 69 years (27.3% [95% CI: 23.6%‐31.3%]) and ages 40 to 59 years (15.7% [95% CI: 12.9%‐19.0%]; Figure 1A). The most common etiology of hearing loss was aging (66.3% [95% CI: 60.8%‐71.4%]) followed by loud long‐term noise (30.3% [95% CI: 26.2%‐34.9%]) and loud brief noise (13.8% [95% CI: 10.3%‐18.4%]; Figure 1B). Overall, 36.8% [95% CI: 32.0‐41.9] reported hearing loss mainly caused by noise exposure.

**Table 1:** Weighted Cohort Characteristics, NHANES 2017 to 2020 (Unweighted n = 797)

Characteristics	Weighted %^a^ (95% CI)
Age	
70‐79 years	59.0 (54.2, 63.8)
80+ years	41.0 (36.3, 45.8)
Sex	
Female	52.1 (46.7, 57.4)
Male	47.9 (42.6, 53.3)
Race/ethnicity	
White	81.1 (75.7, 85.6)
Black	6.0 (3.8, 9.4)
Hispanic	6.3 (4.5, 8.6)
Asian	3.3 (1.9, 5.7)
Other	3.3 (1.8, 6.0)
Education	
Less than high school	14.7 (11.8, 18.1)
High school graduate	33.4 (29.8, 37.4)
Some college or more	51.8 (47.6, 56.0)
Income to poverty ratio	
<1	5.0 (3.6, 6.9)
1 to <4	52.4 (44.2, 60.4)
4 or Greater	29.5 (22.3, 38.0)
Unknown^b^	13.1 (9.6, 17.7)
Health insurance	
No	2.1 (1.1, 4.0)
Private	2.1 (1.2, 3.5)
Medicare	93.8 (90.9, 95.8)
Unknown^b^	2.1 (1.2, 3.5)
Work exposure to loud noise	
Yes	39.2 (33.7, 45.0)
No	59.7 (54.6, 64.6)
Recreational exposure to loud noise	
Yes	9.8 (7.2, 13.2)
No	90.2 (86.8, 92.7)

Work exposure to loud noise was defined as answering yes to: “Have you ever had a job or combination of jobs where you were exposed to loud sounds or noise for 4 or more hours a day, several days a week?” Recreational exposure to loud noise was defined as answering yes to: “Outside of a job, have you ever been exposed to very loud noise or music for 10 or more hours a week?”

^a^ Survey weights were applied according to the National Health and Nutrition Examination Survey; percentages were based on weighted survey sample.

^b^ Unknown includes following responses: “Refused,” “Missing,” and “Don't Know.”

## Discussion

In this study, we assessed the self‐reported onset and etiology of hearing loss among older adults in the United States. To our knowledge, this is the first study to present an overview of hearing loss by age of onset and etiology based on a nationally representative sample.

The prevalence of hearing loss incorporating both self‐report and audiometric data has been extensively examined in the United States.^11^, ^12^ Recent studies have demonstrated a detailed profiling of audiometric characteristics and hearing aid utilization among older adults.^13^ However, the onset and etiology of hearing loss among older adults has not been reported on a population‐level. This study provides overview of the self‐reported onset and etiologies of hearing loss among older adults, providing insights into when older adults become aware of their hearing loss and what they attribute it to. While this data lacks clinical validation regarding the reported onset and etiologies of hearing loss, which likely affects accuracy,^14^ it offers insights into the age at which older adults notice hearing loss and their understanding of its main causes on a population level.

The majority of individuals self‐reported onset of hearing loss later in life, with 69.0% reporting hearing loss onset at age 60 or greater. Recent US Preventive Services Task Force recommendations included an “Insufficient Evidence” grade on a recommendation for hearing loss screening in adults 50 and older.^8^ While the onset of audiometry‐measured hearing loss is unavailable in this data—which is potentially earlier than when older adults start noticing hearing loss—our findings suggest the need to explore the benefits of hearing screening both at an earlier age, before older adults notice hearing loss, versus later in life, such as at ages 60 or 70, when older adults themselves begin to notice hearing loss.

In the United States, the most reported etiology of hearing loss among older adults following aging (66.3%) was related to noise exposure (36.8%). Hearing loss remains among the most common occupational hazard worldwide,^15^, ^16^ while exposure to recreational noise from personal devices and music venues is prevalent, particularly among younger adults.^17^, ^18^ This demonstrates that a large portion of hearing loss can be potentially reduced, highlighting an area of focus for interventions. While Occupational Safety and Health Administration regulations regarding workplace hearing protection and Center for Disease Control and Prevention recommendations for recreational noise exposure exist,^19^, ^20^ the data presented here demonstrates room for improvement in regulations on noise exposure. Future research and interventions should explore effective strategies for mitigating known etiologies of hearing loss, such as noise exposure, infections, injuries, and the adverse effects of certain drugs and medications.^21^

This study is limited by use of self‐reported presence, onset and etiology of hearing loss, and lack of data on temporal changes. Nevertheless, this study provides insights into the prevalence of onset and etiologies of hearing loss among older adults, contributing to our understanding of the potential impact of hearing screening and intervention strategies in the United States. Future studies may explore the differential impact of hearing loss onset and etiology on negative health outcomes known to be associated with hearing loss, including psychosocial and cognitive outcomes.

## Author Contributions

**Tyler J. Gallagher**, conception and design of work, data acquisition and analysis, interpretation of data, drafting of manuscript, critical revision; **Ziphron Russel**, interpretation of data, drafting of manuscript, critical revision; **Janet S. Choi**, Conception and design of work, data acquisition and analysis, interpretation of data, drafting of manuscript, critical revision.

## Disclosure

### Competing interests

None.

### Funding

None.
